# Evaluation of heavy metal concentration in drinking water, feed, and milk of dairy cows in Kombolcha metropolitan town, Ethiopia

**DOI:** 10.1016/j.isci.2026.115001

**Published:** 2026-02-18

**Authors:** Mohammed Yimer, Abebe Bereda, Aleme Asresie

**Affiliations:** 1 Department of Dairy Processing Technology, Federal Technical and Vocational Training Institute, Addis Ababa, Ethiopia; 2 Department of Animal Sciences, Wollo University, Dessie, Ethiopia

**Keywords:** Environmental science, Environmental health, Applied sciences

## Abstract

Cadmium (Cd), chromium (Cr), and lead (Pb) are significant industrial pollutants that contaminate soil, water, plants, and food, posing severe health hazards. Chromium, in particular, is associated with respiratory, kidney, and reproductive issues. In Kombolcha, untreated industrial wastewater increasingly threatens agricultural productivity and water quality. This study examined heavy metal contamination in dairy cows' feed, drinking water, and milk. A cross-sectional survey and laboratory tests using a flame atomic absorption spectrophotometer analyzed 40 samples from water sources, feed, and milk. Water samples showed mean Pb levels of 0.0205 ± 0.004 mg/L and Cd at 0.0112 ± 0.0029 mg/L, with Cr below detection. In elephant grass, Cd was 1.47 ± 0.0016 mg/kg; Cr 1.87 ± 0.115 mg/kg; and Pb 11.4 ± 0.0031 mg/kg. While Cr and Pb in hay and brewery grain were within safe limits, levels in elephant grass and some milk samples exceeded international safety standards. The findings highlight the vital need to improve wastewater treatment and strengthen health protections.

## Highlights

• Brewery by-products and hay are the main feed for dairy cattle
• 40% of major industries dump untreated wastewater into the Borkena river
• Borkena reservoirs had higher cadmium; Ras Anges had higher lead levels
• Cd and Pb found in water, feed, milk; Cr below detection limits

## Graphical abstract

**Figure:**
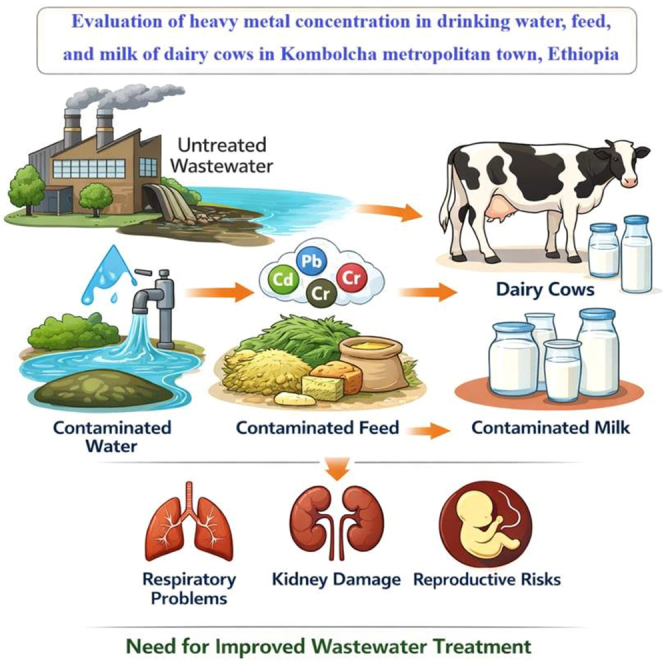

## Introduction

In developing countries such as Ethiopia, rapid industrial growth, urban expansion, and intensified agricultural activities have led to the discharge of large amounts of waste containing hazardous pollutants, particularly heavy metals. These pollutants accumulate in the environment, contaminating air, water, and soil, and can ultimately enter the food chain through grazing animals and crop production.^1^ As industrialization and urbanization expand, the risk of food contamination by toxic metals also increases. Sources such as irrigation water, polluted soils, farming practices, air emissions, and contaminated animal feed further contribute to the transfer of heavy metals into food products.^2^ Mitigation strategies are vital for reducing contamination risks associated with heavy metals. Implementing proper industrial waste treatment, monitoring water quality, and utilizing uncontaminated feed sources are fundamental steps. Additionally, treating wastewater and solid waste before disposal is crucial to prevent the release of heavy metals into the environment. Techniques such as adding amendments, such as lime, phosphates, or biochar, can immobilize heavy metals, decreasing their bioavailability. For instance, phosphate amendments can precipitate metals as insoluble phosphates, effectively limiting their mobility and environmental impact.^3^^,^^4^

Bioremediation offers environmentally friendly solutions for contaminated sites. Hyperaccumulating plants such as *Brassica juncea* and *Sedum alfredii* have shown significant potential for extracting heavy metals from soil and water. In cases of severe contamination, methods such as excavation and soil washing are employed, though these can be costly and disruptive. Microbial remediation, involving bacteria and fungi that metabolize or sequester metals, also shows promise, especially when specific microbial strains are used for bioaugmentation in sediments and soils.^5^ Combining physical, chemical, and biological methods tailored to site conditions, supported by regular environmental monitoring and advanced technologies such as nanomaterials and genetically modified plants, can optimize cleanup efforts and promote sustainable management of heavy metal contamination.

Milk and dairy products, while highly nutritious and vital for human health, particularly for children, are still susceptible to contamination. They can carry residues of harmful biological and chemical substances that threaten consumer health.^6^^,^^7^^,^^8^ Biological contamination is commonly linked to poor milking hygiene, including exposure of udders to environmental pollutants and the use of unclean equipment and storage containers.^9^^,^^10^ Chemical contamination arises from the use of veterinary drugs, contaminated feed, agrochemicals, and improper handling during milk production, processing, and storage.^11^

Studies show that the most commonly reported milk contaminants include heavy metals (22.18%), antibiotics (22.18%), pesticides (22.05%), pathogenic microorganisms (14.57%), and mycotoxins (9.97%), with heavy metals standing out as particularly concerning.^10^^,^^11^^,^^12^ While essential minerals such as iron (Fe), zinc (Zn), and copper (Cu) are beneficial in small amounts, toxic metals, such as lead (Pb), cadmium (Cd), mercury (Hg), chromium (Cr), and arsenic (As) are harmful even at low concentrations.^10^ These metals are linked to a range of health issues, including damage to the nervous system, kidneys, cardiovascular system, and even cancer.^13^^,^^14^ Vulnerable groups such as children, pregnant women, and immunocompromised individuals are especially at risk.^12^

According to the World Health Organization (WHO) and the Food and Agriculture Organization of the United Nations (FAO), food safety is a science-based practice aimed at preventing the presence of harmful substances in food.^2^ As the concentration of heavy metals increases, both the safety and nutritional quality of milk decline significantly.^11^

In Kombolcha metropolitan town, located in Ethiopia’s Amhara region, industrial activities have reportedly discharged untreated wastewater into nearby farmlands and rivers. This environmental pollution raises serious concerns about the safety of local dairy products. Despite this, there is limited empirical evidence on the levels of heavy metals present in drinking water, animal feed, and milk produced in this area.

Therefore, assessing the extent of heavy metal contamination is essential for guiding local interventions such as water treatment, safe feed management, and hygiene practices. This study seeks to fill this critical knowledge gap by evaluating the concentrations of toxic heavy metals in dairy cow feed, drinking water, and milk in Kombolcha metropolitan town, with the ultimate goal of ensuring public health and food safety.

### Study areas

The study was conducted in Kombolcha metropolitan town, located in the South Wollo Zone of the Amhara National Regional State, Ethiopia, which hosts numerous large textile, steel, plastic, brewery, and tannery factories. Geographically, the town lies at a latitude of 11°5′12″ N and a longitude of 39°44′12″ E, with an elevation ranging from 1,842 to 1,915 m above sea level.^7^ Kombolcha has a total population of 125,654, consisting of 62,852 men and 62,802 women. It is situated approximately 376 km northeast of Addis Ababa, along the main route to Mekelle, Tigray region. The area receives an annual rainfall between 750 mm and 900 mm, and the average temperature typically ranges from 25°C to 30°C. According to,^8^ there are 58 dairy farms operating in the town. Kombolcha is also known for hosting one of the highest concentrations of industrial activities in the Amhara region.

## Results

### Major dairy cow feed and drinking water resources

The primary feed resources used by dairy farmers in the study area are presented in Figure S1. The predominant sources included agro-industrial byproducts, particularly brewery spent grains and bean bran, as well as hay and improved forages such as elephant grass. Among the agro-industrial byproducts, brewery spent grains (27.6%) and bean bran (19.0%) were the most commonly reported feed types. Besides, hay (41.4%) and elephant grass (12.1%) were widely used forage resources for dairy cattle.

Tap water was identified as the sole source of drinking water for dairy animals across all farms, reflecting the intensive production systems practiced in the study area. Brewery spent grains were primarily obtained from brewery outlets of BGI Ethiopia, while bean bran was sourced through individual distributors. Elephant grass was locally cultivated by the farmers, whereas hay was typically purchased from other nearby areas. Overall, preserved hay, improved elephant grass, and agro-industrial byproducts formed the core feed base for dairy cattle in the study area.

### Status of the industrial effluent water treatment in the town

The status of industrial wastewater treatment implementation in the study area is shown in Figure S2. Among the large-scale industries assessed, 60% were found to have functional wastewater treatment systems and were actively managing their effluents. Conversely, 40% of the industries lacked any form of treatment and discharged untreated wastewater directly into the Borkena river. This practice poses significant environmental and public health risks, highlighting a critical gap in industrial waste management compliance.

### Toxic heavy metals in dairy cow drinking water, feed, and milk

#### Toxic heavy metal concentrations in dairy cattle drinking water

The levels of toxic heavy metals found in the drinking water of dairy cattle from two reservoir sources are shown in Table S7. Borkena exhibited a higher cadmium concentration (0.02 ± 0.0016 mg/L) compared to Ras Anges (0.0042 ± 0.0024 mg/L). The overall average concentration for both sites is 0.0112 ± 0.0029 mg/L, reflecting differences between the locations. Both reservoirs had chromium levels below the detection limit (<LOD), indicating minimal or no chromium contamination. Ras Anges showed a slightly elevated lead level (0.022 ± 0.0072 mg/L) relative to Borkena (0.019 ± 0.0012 mg/L); with an average lead concentration of 0.0205 ± 0.0042 mg/L, suggesting comparable contamination levels. Overall, Borkena had higher cadmium levels, whereas Ras Anges had slightly higher lead concentrations. The absence of detectable chromium in both sites underscores the importance of continuous monitoring to evaluate potential environmental and health risks associated with heavy metal exposure.

#### Toxic heavy metal concentration in dairy cattle feed

The concentration of heavy metals found in four major dairy cattle feed are shown in Table S8. The results indicate variations in heavy metal concentrations across different feed types. Hay showed relatively low levels of cadmium (Cd), chromium (Cr), and lead (Pb), with concentrations of 0.125 ± 0.0014 mg/kg, 0.6995 ± 0.014 mg/kg, and 0.685 ± 0.00085 mg/kg, respectively. Elephant grass exhibited significantly higher concentrations of all metals, with Cd at 1.47 ± 0.0019 mg/kg, Cr at 1.87 ± 0.115 mg/kg, and Pb at 11.4 ± 0.0096 mg/kg, indicating a marked increase compared to hay. Brewery grains contained low levels of Cd (0.133 ± 0.0017 mg/kg) and Pb (0.9 ± 0.0077 mg/kg), but chromium was below the limit of detection (LOD). Bean bran showed undetectable levels of all three metals, suggesting minimal contamination. Overall, elephant grass demonstrated the highest accumulation of heavy metals, which could pose potential risks if used extensively as feed.

### Concentrations of toxic heavy metals in fresh milk samples

The average concentrations of the selected toxic heavy metals in fresh whole milk samples from four different sites are presented in Table S9. Abisha Ager displayed the highest mean cadmium (Cd) level at 0.03597 ± 0.0157 mg/L, which was statistically comparable to Birarro (0.02275 ± 0.01539 mg/L) and Borkena (0.02079 ± 0.022 mg/L). Hassan Ager had the lowest Cd concentration at 0.010 ± 0.01315 mg/L, significantly lower than the other sites. For chromium (Cr), Abisha Ager also recorded the highest mean level at 0.2952 ± 0.1632 mg/L, followed by Birarro (0.2305 ± 0.0623 mg/L) and Borkena (0.1687 ± 0.1197 mg/L), with no significant differences among these locations. Hassan Ager showed Cr levels below the detection limit (<LOD), indicating minimal or undetectable Cr presence. Regarding lead (Pb), Abisha Ager had the highest mean concentration at 0.2583 ± 0.2495 mg/L. Birarro’s Pb level was significantly lower at 0.0308 ± 0.0123 mg/L, lower than Abisha Ager but not significantly different from Borkena, which measured 0.02776 ± 0.014 mg/L. Hassan Ager again had the lowest Pb concentration at 0.0182 ± 0.00989 mg/L. Overall, Abisha Ager exhibited higher levels of Cd, Cr, and Pb compared to the other sites. The absence of detectable Cr in Hassan Ager suggests lower contamination levels for Cr at this location. These findings highlight notable differences in heavy metal contamination across the sampled sites.

## Discussion

Hay, elephant grass, brewery spent grains, and bean bran were reported to be the major feed sources. The present study confirmed that hay, improved forages (e.g., elephant grass), and agro-industrial byproducts remain the primary feed resource in urban dairy cattle systems in Ethiopia, aligning closely with findings from previous empirical studies. For instance,^15^ documented extensive use of brewery byproducts, wheat bran, and other locally available concentrates around the Addis Ababa and Holetta areas, confirming their dominance as supplemental feeds in urban dairy systems similarly,^16^ identified agro-industrial byproducts as dominant feeds in urban dairy farms in Dire Dawa and Harar cities, reinforcing patterns of industrial feed reliance across geographical contexts.

A study conducted in the districts of Ada’a, Sululta, and Debre Berehan, which is located near a major brewery factory, found that wet brewery spent grain (WBSG) is widely utilized as a feed resource among urban and peri-urban dairy producers. Specifically, over 69% of the 160 dairy farmers surveyed reported incorporating WBSG into their feeding practices, often by mixing it with other feed types such as concentrates and roughages. Despite its widespread use, farmers noted several constraints, including irregular supply, susceptibility to spoilage (e.g., mold and foul odor), and concerns over potential health risks to livestock. Contrastingly, in west Shewa zone, byproducts such as brewery byproducts and improved fodder were observed to be less utilized, largely due to limited supply and lower farmer awareness.^17^

Tap water was the sole source of drinking water for all dairy cattle, reflecting dependency on municipal water supplies. Likewise,^18^ reported that 64% of the urban dairy farmers used municipal water for both household and animals. Although this shows the heavy reliance of urban dairy systems on city water, it must be regularly monitored for potential contaminants, such as heavy metals, that could affect animal health and milk quality.

About 40% of industries in Kombolcha discharged untreated wastewater into the Borkena river, posing environmental and public health risks. Similar trends have been reported in Addis Ababa, where industrial effluent management is inadequate, contaminating surface water with heavy metals such as Cr and Zn.^19^ Effluent from breweries and tanneries contributes significantly to water and soil contamination, threatening dairy production.^20^

The average concentrations of cadmium (Cd) and lead (Pb) in the analyzed water samples exceeded the permissible limits established by international guidelines (0.003 mg/L for Cd and 0.01 mg/L for Pb).^21^^,^^22^^,^^23^^,^^24^ Notably, the Borkena reservoir exhibited higher Cd levels than Ras Anges, likely due to inadequate water treatment and potential pipeline corrosion, a recognized source of metal leaching into water systems.^25^^,^^26^

In the present study, the mean Cd concentration was 0.0112 ± 0.0029 mg/L, comparable to levels reported in Mekelle (0.017 mg/L),^27^ but higher than those documented in Addis Ababa (0.002 mg/L),^28^ Assela (0.005 mg/L),^29^ and 0.0062 ± 0.01 ppm.^30^ Conversely, it was lower than concentrations reported in other regions, including 0.359 ± 0.026 mg/L,^31^ 0.816 mg/L,^32^ and 0.12 mg/L.^33^ The mean Pb concentration was 0.0205 ± 0.0042 mg/L, similar to that reported in Addis Ababa (0.028 mg/L),^22^ but lower than other studies verifying 0.660 ± 0.064 mg/L,^25^ 0.04 mg/L in Assela,^23^ and 5.2 mg/L elsewhere.^26^ These variations likely reflect differences in water treatment efficiency, contamination sources, and infrastructure integrity, underscoring the potential health risks of Cd and Pb exposure to both humans and livestock.^28^

Elephant grass used as cattle feed was found to contain cadmium (Cd) and lead (Pb) at levels exceeding internationally recommended safety limits (Cd: 1 mg/kg; Pb: 10 mg/kg) [30].^34^ These high levels are likely linked to irrigation with polluted water and airborne contamination from nearby industrial activities. In contrast, other feed types such as hay, brewery spent grains, and bean bran, which are less exposed to industrial effluents, remained within safe limits.

This pattern of contamination is consistent with previous studies showing elevated Cd, Pb, and chromium (Cr) levels in wastewater, soil, and surface water downstream of industrial discharge points along the Borkena river.^35^^,^^36^ Looking at global comparisons, the Cd level in elephant grass (1.47 mg/kg) was higher than in maize fodder from uncontaminated areas of Pakistan, teff straw from Holetta, and roadside grasses in Botswana,^37^^,^^38^ but lower than concentrations reported in cattle feed from China and Turkey.^35^^,^^39^^,^^40^ Such differences reflect how environmental conditions and proximity to industry influence feed safety.

Chromium and lead followed similar trends. Chromium in conserved hay (0.70 mg/kg) was comparable to previous reports but lower than in elephant grass (1.87 mg/kg), though still below extreme levels found in teff straw from polluted regions.^37^^,^^41^^,^^42^ Lead in elephant grass (11.4 mg/kg) was notably higher than in most other feeds, although it remained below levels reported in heavily contaminated fodder in Pakistan.^37^^,^^43^ These findings highlight the significant impact of industrial activity on feed safety, especially for elephant grass. Cadmium, in particular, is a known human carcinogen linked to lung and other cancers, underscoring the importance of monitoring livestock feed and reducing exposure to environmental pollutants. Experiences from Pakistan, China, and Turkey confirm that industrial proximity consistently increases heavy metal contamination in fodder.^37^^,^^39^

In this study, statistically significant variations in cadmium (Cd) and lead (Pb) concentrations in fresh whole milk were observed, likely linked to the contamination of dairy cattle feed, particularly elephant grass irrigated with wastewater and polluted water sources. The Borkena river basin has been previously reported to experience compromised soil, pasture, and irrigation water quality due to industrial effluent discharge.^35^

The mean Cd concentration in milk samples was 0.03597 ± 0.0157 mg/L, exceeding the permissible limit of 0.0026 mg/L at all sampling sites.^44^ Similarly, Pb concentrations exceeded the recommended threshold of 0.02 mg/L, with the highest levels recorded in Abisha Ager (0.2495 ± 0.2583 mg/L), followed by Birarro (0.0308 ± 0.0123 mg/L) and Borkena (0.02776 ± 0.014 mg/L). In contrast, chromium (Cr) levels (0.2952 ± 0.1632 mg/L) remained below the recommended limit of 0.3 mg/L.^44^

These elevated Cd and Pb levels can be attributed to environmental exposure pathways, including contaminated irrigation water, industrial wastewater, and atmospheric deposition of airborne pollutants. Evidence from Kombolcha shows that both treated and untreated effluents from local breweries, tanneries, and textile factories contain heavy metals,^45^ while Cd and Pb are recognized as common airborne pollutants from industrial activities.^46^

When compared to other studies, the Cd levels observed in this study are consistent with reports from cow milk in Bangladesh (0.03 mg/L) and Ethiopia (0.0359 mg/L), respectively.^47^^,^^48^ However, they exceed values reported in Laygaiynt district (0.0045 mg/L),^49^ Addis Ababa (0.01 mg/L),^50^ and cow milk (0.0186 mg/L),^51^ yet remain lower than those from Debre Tabor (0.0927 mg/L).^52^ Cr concentrations in milk were comparable to Holeta (0.13974 mg/L) but lower than reports from Uganda (6.84 ± 2.03 mg/L) and milk with Cr up to 31.4 ppm, respectively,^37^^,^^53^ likely reflecting feed and water contamination near industrial zones.

Pb levels in Abisha Ager were consistent with earlier reports in raw milk (0.263 mg/L), though lower than cow milk from Iran (12.52 ± 1.78 mg/L) and other high-exposure regions, respectively.^54^^,^^55^ Nonetheless, these values exceeded levels reported in Laygaiynt (0.00284 mg/L) and in studies from Uganda and Libya.^50^^,^^54^

Overall, these findings indicate that industrial emissions, wastewater irrigation, and traffic-related pollutants have infiltrated feed and water sources, resulting in elevated Cd and Pb levels in milk. Such contamination poses significant public health risks, as Cd and Pb are known carcinogens and neurotoxins capable of bioaccumulation in both animals and humans.^55^^,^^56^ Milk from Abisha Ager showed Cd (0.03597 mg/L) and Pb (0.2495 mg/L) exceeding permissible limits, while Cr (0.2952 mg/L) remained below the threshold,^57^ confirming contamination originating from feed and water sources near industrial discharge points.^58^^,^^59^

### Conclusion

The study detected cadmium (Cd) and lead (Pb) in water samples, while chromium (Cr) was below the detection limit. In feed, Cd, Cr, and Pb were present in elephant grass and hay; brewery grains contained Cd and Pb, and bean bran showed no detectable contamination. Some water and feed samples exceeded international safety limits for Cd and Pb. Elevated Cd and Pb levels in milk suggest potential transfer from contaminated feed and water, though the absence of correlation analysis limits the confirmation of specific contamination pathways.

Overall, the findings underscore the potential risks of heavy metal contamination in dairy production in Kombolcha. Future research should aim to increase the sample size for feed and water samples associated with milk, employ more rigorous analytical methods, and ensure proper calibration of analytical instruments. Additionally, correlation, regression, or multivariate analyses were not conducted in this study to examine the relationships between heavy metal levels in water, feed, and milk. Incorporating these analytical approaches in future studies will provide a clearer understanding of the pathways and interactions of heavy metal contamination among water, feed, and milk in dairy cattle.

### Limitations of the study

The limitations of this study include a relatively small sample size of feed, milk, and water, as well as the analysis of only three heavy metals, which may restrict the generalizability of the findings. Additionally, the absence of advanced statistical analyses such as regression or multivariate techniques, hindered a thorough understanding of the relationships and contamination pathways among water, feed, and milk. These limitations indicate that future research should involve larger sample sizes, more comprehensive analytical methods, and sophisticated data analysis to better clarify the extent, sources, and mechanisms of heavy metal contamination in dairy production.

## Resource availability

### Lead contact

Further information and other requests should be directed to and will be fulfilled by the lead contact, Aleme Asresie (aleme.asres@wu.edu.et).

### Materials availability

This work did not generate new unique reagents. All the materials and methods used for the generation of data and analysis are mentioned in the article.

### Data and code availability

• All data reported in this article will be shared by the lead contact upon request.
• This article does not report original code.
• Any additional information required to reanalyze the data reported in this article is available from the lead contact upon request.

## Acknowledgments

The authors express their gratitude to 10.13039/501100007941Addis Ababa University and the Federal Technical and Vocational Training Institute, Ethiopia for providing the facilities and financial support necessary to complete this work. They also extend their appreciation to the data collectors, key informants, focus group participants, and respondents who contributed valuable information.

## Author contributions

M. Y., A. B., and A. A. contributed to the conceptualization, investigation, methodology, original draft writing, review and editing, and formal analysis of the study. A. B. and A. A. also provided supervision.

## Declaration of interests

The authors declare no competing interests.

## STAR★Methods

### Key resources table

**Table:** 

REAGENT or RESOURCE	SOURCE	IDENTIFIER
**Biological samples**		

30 milk samples	The Kombolcha metropolitan smallholder dairy farmers	N/A
3 composite hay samples	The Kombolcha metropolitan town smallholder dairy farmers	N/A
3 composite elephant grass samples	The Kombolcha metropolitan town smallholder dairy farmers	N/A
1 brewery grain sample	BGI Ethiopia based in Kombolcha metropolitan town	N/A
1 bean bran sample	The Kombolcha metropolitan town local feed distributors	N/A
2 water samples	The Kombolcha metropolitan town water reservoirs of Ras Anges and Borkena	N/A
58 dairy farms	The Kombolcha metropolitan town smallholder dairy farmers	N/A
10 industries	The Kombolcha metropolitan town	N/A

**Chemicals**		

HNO_3_	National Library of Medicine	Cat# *84380*
H_2_O_2_	National Library of Medicine	Cat# 216763
Distilled water	Adis Ababa Analytical Chemistry Laboratory	N/A

**Software and algorithms**		

SPSS	Liang et al.^60^	http://www.spss.com.hk/

**Other**		

Flame Atomic Absorption Spectro photometer(FAAS)	NovAA 350, Analytik Jena, Germany	N/A
Muffle furnace	Harrier Enterprises	N/A
Whatman Grade 1 qualitative filter paper	Cytiva	Cat# 1001-110
Polyethylene sampling bottles	Commercially available	Cat# 2087079
Stainless steel knives	Commercially available	N/A
Analytical balance	China Laboratory KOOKYOU	Cat# WTC 200
Volumetric flasks	Hirschman Laboratory GmbH &Co.Kg	N/A
Beakers	Commercially available	N/A
Conical flasks	Commercially available	N/A
Graduated cylinders	Commercially available	N/A
Pipettes	Commercially avaliable	N/A
Refrigerator	Electrolux	N/A

### Experimental model and study participant details

There are no experimental models (animals, human participants, plants, microbe strains, cell lines, primary cell cultures) used in the study.

### Method details

#### Study design

This study used both cross sectional surveys and laboratory analyses to assess heavy metal contamination in dairy production. Surveys, Key informant interviews (KIIs), Focus group discussions (FGDs), and interviews were conducted with stakeholders, and observations were made at farms and industries to gather information on feed, water, and wastewater practices. Laboratory tests were then carried out on water, feed, and milk samples to determine heavy metal concentrations and Analysed in Addis Ababa University.

#### Sampling techniques and sample size determination

The study area, Kombolcha metropolitan town, contains 58 registered dairy farms and ten major industrial facilities, including textile, steel, plastic, brewery, and tannery factories. Because the number of farms and industries was manageable, a census approach was used to include all of them in the survey. This ensured comprehensive coverage of potential contamination sources and improved the accuracy of the assessment of heavy metal risks in the local dairy production system.

All 58 farms participated in the survey regarding the major feed resources used; however, due to budget constraints, only 30 farms were selected for milk sample collection and analysis of heavy metal contamination. During the survey, all cattle feed sources were identified, and the major ones hay, elephant grass, brewery grains, and bean bran were selected for laboratory testing. Hay was sampled from 12 farms, chopped, mixed, and pooled into three composite samples. Elephant grass was collected from six farms using quadrats along diagonal transects, with grass harvested from five positions in each quadrat and combined into three representative samples. Brewery grains were obtained from the BGI Ethiopia (an Ethiopian Brewery and Beverage Company) and it is one of the livestock brewery grain feed resource, while bean bran was purchased from a local feed distributor. Drinking water used for dairy cattle was collected from both the Ras Anges and Borkena reservoir sources for analysis.

#### Methods of data collection

Data collection for this study involved both primary and secondary sources. Primary data were gathered through semi-structured questionnaires administered to dairy farm owners and representatives of large factories. Additional insights were obtained through eight focus group discussions (FGDs) and four key informant interviews (KIIs) with development agents, senior agricultural experts, and managers from farms and industries. Direct field observations were also conducted to verify information, focusing on the major feed and water sources for dairy cattle and the condition of industrial wastewater discharge and treatment systems.

Secondary data were obtained from relevant literature, national and international guidelines, reports from the Kombolcha town livestock and fisheries resource development office, and annual reports from the selected factories. To complement the survey data, laboratory analyses were performed to assess the concentrations of selected heavy metals in dairy cow feed, drinking water, and milk, providing a comprehensive evaluation of potential contamination sources and associated health risks.

#### Chemicals and reagents

All chemicals and reagents used in this study were of analytical grade and were selected to ensure accurate determination of heavy metals in milk, feed, and water samples collected from Kombolcha metropolitan town, an area characterized by intensive industrial activities that may contribute to environmental contamination.

Feed samples were dried in a muffle furnace at 600°C and then weighed at 5 grams. They were subsequently subjected to wet digestion by adding 5 mL of 33% nitric acid (HNO_3_) and 10 mL of 50% hydrogen peroxide (H_2_O_2_), followed by heating at 100°C for one hour. The digested samples were filtered using filter paper. The resulting solutions were nebulized into flame atomic absorption spectrophotometer (FAAS) to determine the concentration levels of lead, chromium, and cadmium in the feed.^61^

Fifty milliliters of water samples were measured into a volumetric flask and digested with 1 mL of 50% H_2_O_2_. The samples were then nebulized into FAAS to determine the concentration levels of lead, chromium, and cadmium in the water.^61^

Fifteen milliliters of each cow’s milk sample were digested by adding 10 mL of 65% nitric acid (HNO3) and 3 mL of 30% hydrogen peroxide (H2O2). The mixture was initially heated at 35–40°C for 30 minutes, then increased to 95°C and maintained for one hour. After digestion, the samples were cooled to room temperature to prevent foaming and splashing. To each sample, 10 mL of distilled water was added, and the mixture was filtered using Whatman Grade 1 qualitative filter paper. The resulting clear filtrate was nebulized into FAAS to determine the concentrations of lead, chromium, and cadmium in the raw cow milk.^61^ Concentrated nitric acid (HNO_3_, 65%), hydrogen peroxide (H_2_O_2_, 30–50%), and deionized water were used for sample digestion, cleaning of sampling containers, and dilution procedures. Stock standard solutions (1000 mg/L) of cadmium (Cd), chromium (Cr), and lead (Pb), prepared in HNO_3_, were used to prepare working standard solutions for calibration of the FAAS. Polyethylene sampling bottles and laboratory glassware were pre-soaked in 20% nitric acid for 24 hours and thoroughly rinsed with deionized water to minimize contamination. Whatman Grade 1 qualitative filter paper was used for filtration of digested samples. All reagents were handled following standard laboratory safety and quality control procedures to ensure reliability of the analytical results.

#### Apparatus

The apparatus used in this study were selected to support reliable sample collection, preparation, digestion, and analysis of heavy metals in milk, feed, and water samples from the Kombolcha metropolitan area. Sample collection materials included acid-washed polyethylene bottles (100–350 mL), stainless steel knives for feed sampling, quadrats for pasture sampling, ice boxes with ice packs, and airtight plastic bags for sample storage and transport. Laboratory equipment comprised a FAAS (Model: NovAA 350, Analytik Jena, Germany) equipped with hollow cathode lamps for cadmium, chromium, and lead, a hot plate with temperature control, a muffle furnace, analytical balance, volumetric flasks, beakers, conical flasks, graduated cylinders, and pipettes. Additional apparatus included a refrigerator for sample preservation at 4°C, filtration units with Whatman Grade 1 filter paper, and a computer system interfaced with the FAAS for data acquisition and analysis (Table S1). All apparatus were thoroughly cleaned and conditioned prior to use to prevent cross-contamination and ensure analytical accuracy.

#### Sample digestion and preparation of analyte solution for FAAS

To ensure accurate determination of heavy metals, milk, feed, and water samples were subjected to appropriate digestion procedures to destroy organic matter and release bound metals into solution prior to FAAS analysis.^61^ Milk samples, which contain a complex organic matrix, were digested by treating 15 mL of raw milk with 10 mL of concentrated nitric acid (65% HNO_3_) and 3 mL of hydrogen peroxide (30% H_2_O_2_). The mixture was heated initially at 35-40°C for 30 minutes and then at 95°C for one hour until a clear solution was obtained. After cooling to room temperature, the digest was diluted with distilled water, filtered through Whatman Grade 1 filter paper, and transferred into clean volumetric flasks to obtain the analyte solution for FAAS measurement.

Feed samples were first air-dried and ashed in a muffle furnace at 600°C to reduce organic content. A 5 g portion of each ashed sample was then digested using a mixture of 5 mL nitric acid (33% HNO_3_) and 10 mL hydrogen peroxide (50% H_2_O_2_). The digestion was carried out on a hot plate at 100°C for one hour with continuous heating until complete dissolution was achieved. The resulting solutions were cooled, filtered, and diluted with deionized water to a known volume to prepare clear analyte solutions suitable for FAAS analysis.

Water samples intended for dairy cattle consumption were prepared by transferring 50 mL of each sample into acid-washed volumetric flasks and adding 1 mL of hydrogen peroxide (50% H_2_O_2_) to aid oxidation of any residual organic matter. The mixtures were gently heated when necessary, allowed to cool, and then directly used as analyte solutions. All prepared analyte solutions were stored in clean polyethylene containers and analyzed for cadmium (Cd), chromium (Cr), and lead (Pb) using FAAS under optimized instrumental conditions.

#### Calibration curve and measurement of metal concentrations

Quantification of cadmium (Cd), chromium (Cr), and lead (Pb) in milk, feed, and water samples was performed using FAAS.^61^ Prior to sample analysis, a series of working standard solutions for each metal were prepared by appropriate serial dilution of certified 1000 mg/L stock standard solutions in nitric acid with deionized water. Blank solutions containing the same acid matrix were also prepared. Each set of standards was aspirated into the FAAS under optimized instrumental conditions, and calibration curves were constructed by plotting absorbance against the corresponding standard concentrations. A strong linear relationship between absorbance and concentration was obtained for all metals, with correlation coefficients (R^2^) greater than 0.997, indicating excellent linearity and instrument performance.

The concentrations of Cd, Cr, and Pb in the digested samples were determined by aspirating the prepared analyte solutions into the FAAS and recording their absorbance values. Metal concentrations were calculated directly from the respective calibration curves generated by the FAAS software (Table S2). Quality assurance was ensured through repeated measurements, analysis of blanks, and adherence to instrument detection limits. The final concentrations of heavy metals in milk, feed, and water samples were expressed in mg/L or mg/kg, as appropriate, based on sample type and dilution factors.

#### Method and instrument detection limits

The sensitivity of the analytical method and the performance of the FAAS were evaluated by determining both the method detection limits (MDLs) and instrument detection limits (IDLs) for cadmium (Cd), chromium (Cr), and lead (Pb) in milk, feed and water (Table S3). Instrument detection limits were established based on the FAAS manufacturer’s specifications and repeated measurements of blank solutions under optimized operating conditions, defined as three times the standard deviation of the blank signal. The obtained instrument detection limits were 0.0017 mg/L for Cd, 0.005 mg/L for Cr, and 0.001 mg/L for Pb, demonstrating adequate sensitivity for trace-level determination of these metals.

Method detection limits (MDLs) were established by processing reagent blanks through the complete digestion and analytical procedures applied to the samples, thereby ensuring that both potential contamination and matrix-related effects were fully incorporated into the detection limit assessment. The MDLs were calculated as three times the standard deviation of the blank measurements multiplied by the appropriate dilution factors The determined detection limits were sufficiently low to allow reliable quantification of Cd, Cr, and Pb in milk, feed, and water samples from the study area, ensuring the suitability of the method for assessing heavy metal contamination associated with dairy production systems. The limit of detection (LODs) is a crucial metric in analytical chemistry and were generally calculated as three times the standard deviation of a series of measurements of a reagent blank (a sample prepared using the same procedure as the actual samples but without the analyte of interest), divided by the slope of the calibration curve. The LODs were sufficiently low to allow reliable quantification of Cd, Cr, and Pb in milk, feed, and water samples from the study area, ensuring the suitability of the method for assessing heavy metal contamination associated with dairy production systems.

#### Validation methods

The recovery of lead (Pb), cadmium (Cd), and chromium (Cr) was evaluated by spiking milk, feed, and water samples with known quantities of their respective standard solutions (Table S4–S6). The added concentrations were carefully selected within the range of 5–7% of the native metal levels to avoid significant alteration of the original sample composition. For milk, 500 μL of mixed standard solutions containing Pb (0.40 mg/L), Cd (0.005 mg/L), and Cr (0.04 mg/L) were added to 3 mL of each milk sample and subjected to the same digestion procedure used for unspiked milk samples. For feed samples, 500 μL of the same mixed standard solutions were added to 5 g of homogenized feed, followed by digestion using the identical acid digestion protocol applied to the original feed samples. For water samples, 500 μL of the standard solutions were added to 50 mL of each water sample and treated following the same preparation and digestion procedure used for unspiked water samples.

After digestion, all spiked samples were diluted to a final volume of 100 mL with deionized water and analyzed using FAAS under the same instrumental conditions applied to the original samples. All recovery experiments were conducted in triplicate, and triplicate readings were recorded for each metal. The percentage recoveries of Pb, Cd, and Cr were then calculated using the standard recovery formula (Equation 1).

(Equation 1) $$\text{Recovery}\;\left(\%\right)=\frac{C_{\text{spiked}-C_{\text{unspiked}}}}{C_{\text{added}}}*100$$

#### Determination of cadmium, chromium and lead

The concentrations of cadmium (Cd), chromium (Cr), and lead (Pb) in milk, feed, and water samples were determined using FAAS (NovAA 350, Analytik Jena, Germany) following a digestion and calibration procedure adapted from standard methods.^61^

Approximately 5 g of each sample was placed in a crucible and carbonized at 100°C on a hot plate. The samples were then ashed in a muffle furnace at 550°C for 2–3 hours until whitish ash was obtained. The ash was dissolved in 5 mL of 6N HCl and evaporated for 2–3 minutes, followed by the addition of 7 mL of 3N HCl and heating until just boiling. After cooling, the solution was filtered through Whatman No. 1 filter paper into a 50 mL graduated flask. The crucibles were washed at least three times with distilled water, and the washings were added to the flask. The extract was cooled and diluted to the 50 mL mark with distilled water.

Working standard solutions of Cd, Cr, and Pb were prepared by serial dilution of 1000 mg/L stock solutions with deionized water to obtain concentrations of 0, 0.25, 0.5, 1.0, and 2.0 mg/L. Blanks were prepared using the same reagents without samples.

The analysis of cadmium, chromium, and lead was performed using a FAAS.^61^ The wavelength and detection limit for each element were optimized for accurate quantification. Cadmium was measured at a wavelength of 288.8 nm with a detection limit of 0.0017 mg/L. Chromium was analyzed at 357.87 nm, with a detection limit of 0.005 mg/L. Lead was determined at 283.31 nm, with a detection limit of 0.001 mg/L. These parameters ensured high sensitivity and reliability in the determination of trace metal concentrations in milk, feed, and water samples. Concentrations of Cd, Cr, and Pb in the samples were calculated from the calibration curves (Equation 2):

(Equation 2) $$\text{Concentration}\;\left(\text{mg}/100g\right)=\frac{\left(a-b\right)*V}{10*W}$$

Where a = concentration of the sample solution (ppm), b = concentration of the blank solution (ppm), V = volume of the extract (mL), and W = weight of the sample (g). Air-acetylene flame was used as the atomization source. All measurements were performed in triplicate to ensure accuracy and reproducibility.

### Quantification and statistical analysis

Descriptive statistics, such as frequency, percentage, mean, standard deviation, and average were analyzed using SPSS version 25 software.^60^ A one-way analysis of variance (ANOVA) was performed to evaluate significant differences in the mean concentrations of heavy metals across in feed, water and milk samples. The Tukey post-hoc test was used to identify specific differences among the toxic heavy metals. A significance level of p < 0.05 was set for all statistical tests.
