# Supplementary material for: Evaluation of heavy metal concentration in drinking water, feed, and milk of dairy cows in Kombolcha metropolitan town, Ethiopia
Source: iScience. 2026 Feb 18;29(3):115001. doi: 10.1016/j.isci.2026.115001 (PMC12961311; doi:10.1016/j.isci.2026.115001)
Supplement: Document S1. Figures S1 and S2 and Tables S1–S9 [file mmc1.pdf]

iScience, Volume *29*

Supplemental information

Evaluation of heavy metal concentration in drinking water, feed, and milk of dairy cows in Kombolcha metropolitan town, Ethiopia

Mohammed Yimer, Abebe Bereda, and Aleme Asresie

SUPPLEMENTAL INFORMATION

Table S1. Instrumental conditions for evaluation of Cd, Cr, and Pb from all samples.

Parameters	Toxic heavy metals		
	Cadmium (Cd)	Chromium (Cr)	Lead (Pb)
Wavelength λ (nm)	288.8	357.87	283.31
Slit width (nm)	1.4	0.2	1.2
Sample energy (eV)	55	68	67
Lamp current (mA)	2	1.5	4

Table S2. Series of working standards and correlation coefficients of the calibration curves for the determination of metals in the samples using FAAS.

Metal analyzed	Concentration of standard working solutions (mg/L)	Correlation coefficient (R^2)	Regression equation
Cadmium	0, 0.25, 0.5, 1, 2	0.9762	0.2952x+0.0351
Chromium	0, 0.25, 0.5, 1, 2	0.9998	0.1634x+0.0025
Lead	0, 0.25, 0.5, 1, 2	0.9976	0.0318x+0.0006

Table S3. Detection and instrument limit of Cd (II), Cr (II), and Pb (II) in milk, feed, and water samples.

Metal analyzed	Method detection limit (µg/L)	Instrument detection limit (mg/L)	Milk sample (mL)	Feed sample (g)	Water sample (mL)
Cadmium	0.5–2	0.0017	< 0.5	< 0.5	< 0.5
Chromium	5–15	0.005	< 5	< 5	< 5
Lead	10–20	0.001	< 10	< 10	< 10

Table S4. The recovery (%) of heavy metals in dairy cows feed.

Metal	Spiked analyte concentration (mg/L)	Calculated analyte concentration (n =4) (mg/kg)	Recovery (%) (n =4)	Overall recovery % (n= 12)
Pb	1.5	1.394	92.933	90.471±2.951
	1	0.872	87.2	
	0.5	0.4564	91.28	
Cr	0.5	0.483	96.6	94.71±1.942
	0.25	0.237	94.8	
	0.125	0.1159	92.72	
Cd	2	1.89	94.5	97.11±2.3719
	1.5	1.487	99.133	
	1	0.976	97.6	

Table S5. The recovery (%) of heavy metals in drinking water of dairy cows.

Metal	Spiked analyte added concentration (mg/L)	Calculated analyte concentration (n =2) (mg/kg)	Recovery (%) (n = 2)	Overall recovery % (n= 6)
Pb	1.5	1.492	99.466	95.998±3.882
	1	0.967	96.7	
	0.5	0.459	91.8	
Cr	2.5	2.30	92	95.422±3.1069
	2	1.924	96.2	
	1.5	1.471	98.066	
Cd	2	1.846	92.3	86.166 ±4.6047
	1.5	1.287	85.8	
	1	0.834	83.4	

Table S6. The recovery (%) of heavy metals in cow milk.

Metal	Spiked analyte concentration (mg/L)	Calculated analyte concentration (n =30) (mg/kg)	Recovery (%) (n = 30)	Overall recovery % (n= 90)
Pb	0.75	0.772	102.933	96.11±6.3399
	0.5	0.475	95	
	0.25	0.226	90.4	
Cr	0.5	0.4645	92.9	89.82±3.7811
	0.25	0.209	85.6	
	0.125	0.1137	90.96	
Cd	1	0.864	86.64	93.08 ±5.775
	0.5	0.489	97.8	
	0.25	0.237	94.8	

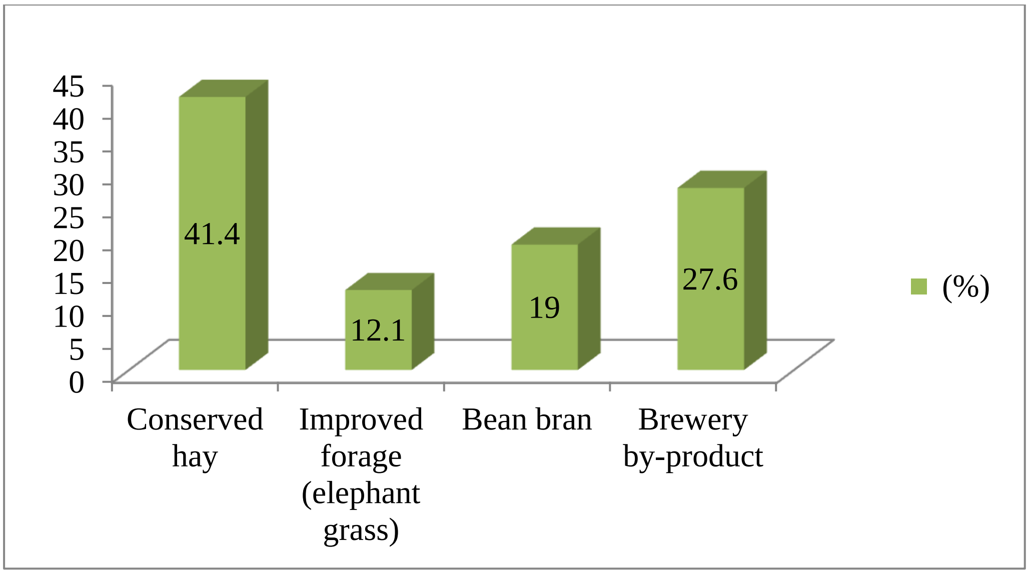

Figure S1. Major feed resource for dairy cows expressed in percentage in Kombolcha town

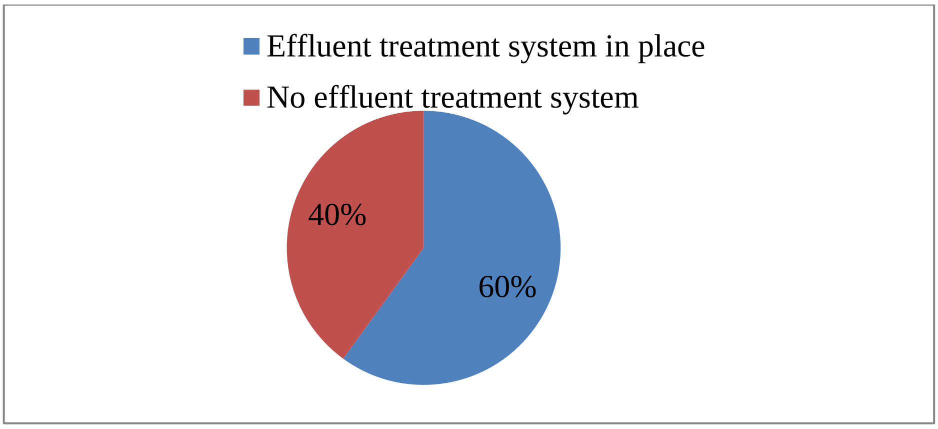

Figure S2. Status of industrial wastewater treatment expressed in percentage in Kombolcha town

Table S7. Average concentration of heavy metals in drinking water samples

Water reservoirs	Concentration of heavy metals (mg/L)		
	Cd	Cr	Pb
Ras Anges	0.0042± 0.0024[a]	<LOD	0.022±0.0072[a]
Borkena	0.02±0.0016[b]	<LOD	0.019±0.0012[a]
Average	0.0112±0.0029	<LOD	0.0205±0.0042

<LOD = less than the limit of detection, means with different superscript between columns were statistically significance ($p<0.05$), the values in the table are Mean ±SD concentration of heavy metals in drinking water of dairy cows.

Table S8. Average concentrations (mg/kg of DM) level of heavy metals in dairy cows feeds

Feed types	Concentration (mg/kg)		
	Cd	Cr	Pb
Hay	0.125±0.0014^{b}	0.6995±0.014^{b}	0.685±0.00085^{b}
Elephant grass	1.47±0.0019^{a}	1.87±0.115^{a}	11.4±0.0096^{a}
Brewery grains	0.133±0.0017^{b}	<LOD	0.9±0.0077^{b}
Bean bran	<LOD	<LOD	<LOD

<LOD=less than the limit of detection, Means with different superscript between columns were statistically significance ($p<0.05$), the values in the table are Mean ±SD concentration of heavy metals in dairy cows feeds.

Table S9. Mean concentrations (mg/L) level of toxic heavy metals in cow's milk

Milk samples sites		Concentration level of toxic heavy metals (Mean ±SD)		
	N	Cd	Cr	Pb
Abisha Ager	10	0.03597±0.0157^{a}	0.2952±0.1632^{a}	0.2583 ±0.2495 ^{a}
Birarro	4	0.02275±0.01539^{ab}	0.2305±0.0623^{a}	0.0308±0.0123^{ab}
Borkena	7	0.02079±0.022^{ab}	0.1687±0.1197^{a}	0.02776±0.014^{b}
Hassan Ager	9	0.010±0.01315^{b}	<LOD	0.0182±0.00989^{b}

<LOD=less than limit of detection, Means with different superscript between columns were statistically significance ($p<0.05$), the values in the table are Mean ±SD concentration of heavy metals in cow's milk.
